# Climate Predicts UV Floral Pattern Size, Anthocyanin Concentration, and Pollen Performance in *Clarkia unguiculata*

**DOI:** 10.3389/fpls.2020.00847

**Published:** 2020-06-16

**Authors:** Kristen Peach, Jasen W. Liu, Susan J. Mazer

**Affiliations:** ^1^Ecology, Evolution, and Marine Biology, University of California, Santa Barbara, Santa Barbara, CA, United States; ^2^Population Biology Graduate Group, University of California, Davis, Davis, CA, United States

**Keywords:** geographic variation, flower color, floral evolution, intraspecific variation, pollen performance, UV patterns, nectar guide

## Abstract

Given that flower size and pigmentation can mediate plant–pollinator interactions, many studies have focused on pollinator-driven selection on these floral traits. However, abiotic factors such as precipitation, temperature, and solar radiation also contribute to geographic variation in floral color, pattern, and size within multiple species. Several studies have described an ecogeographic pattern within species in which high temperature, high ultraviolet (UV) radiation, low precipitation and/or low latitudes are associated with increased floral anthocyanin production, smaller flowers, and/or larger UV-absorbing floral patterns (nectar guides or bullseyes). However, latitude or elevation is often used as a proxy variable to study variation in floral traits associated with a wide range of climatic variables, making the proximate abiotic drivers of variation difficult to identify. In this study, we tested and corroborated several predictions for how the abiotic environment may directly or indirectly shape geographic patterns of floral color, pattern, and size in *Clarkia unguiculata* (Onagraceae). This study provides the first report of geographic variation in multispectral floral color and pattern in *C. unguiculata*, while also providing an experimental test of the putative protective role of UV absorption for pollen performance. We quantified geographic variation among greenhouse-raised populations in UV floral pattern size, mean UV petal reflectance, anthocyanin concentration, and petal area in *C. unguiculata* across its natural range in California and, using 30 year climate normals for each population, we identified climatic and topographic attributes that are correlated with our focal floral traits. In addition, we examined pollen performance under high and low UV light conditions to detect the protective function (if any) of UV floral patterns in this species. Contrary to our expectations, the nectar guide and the proportion of the petal occupied by the UV nectar guide were largest in low solar UV populations. Estimated floral anthocyanin concentration was highest in populations with high solar UV, which does support our predictions. The size of the UV nectar guide did not affect pollen performance in either of the light treatments used in this study. We conclude that, under the conditions examined here, UV-absorbing floral patterns do not serve a direct “pollen protection” function in *C. unguiculata*. Our results only partially align with expected ecogeographic patterns in these floral traits, highlighting the need for research in a wider range of taxa in order to detect and interpret broad scale patterns of floral color variation.

## Introduction

Geographic variation in floral color, pattern, and size is well documented in multiple plant species (Streisfeld and Kohn, 2005; Schemske and Bierzychudek, 2007; Arista et al., 2013; Hopkins and Rausher, 2014; del Valle et al., 2015; Sobral et al., 2015; Tripp et al., 2018). Given that flower size and pigmentation can mediate plant–pollinator interactions (Brunet, 2009; Grossenbacher and Stanton, 2014; Koski and Ashman, 2014; Muchhala et al., 2014), many studies have focused on pollinator-driven selection on these floral traits (Rausher, 2008; van der Niet and Johnson, 2012; Grossenbacher and Stanton, 2014; Muchhala et al., 2014). While it is clear that pollinators, and other biotic factors such as florivores, have shaped floral evolution (see van der Niet and Johnson, 2012 for a review), abiotic factors such as UV (ultraviolet) radiation, temperature and precipitation may also drive intraspecific variation in floral color and form (Arista et al., 2013; del Valle et al., 2015; Campbell and Powers, 2015; Koski and Ashman, 2016; Tripp et al., 2018).

Precipitation, temperature, and solar (UV) radiation are associated with variation in floral color within multiple taxa (Schemske and Bierzychudek, 2001 [*Linanthus parryae*]; del Valle et al., 2015 [*Silene littorea*]; Koski and Ashman, 2015 [*Argentina anserina*]). For example, high temperatures and low water availability are sometimes associated with a higher proportion of pink, purple, or blue floral color morphs compared to white ones (Schemske and Bierzychudek, 2001; Berardi, 2014). Anthocyanins (which commonly confer orange, red, blue, or purple coloration to plant tissues) may also provide protection against environmental stressors such as extreme (high or low) temperatures, low water availability, high UV light, or other abiotic factors (Lee and Gould, 2002; Lee and Finn, 2007; Landi et al., 2015). In flower buds, anthocyanins may help to protect developing reproductive tissues from antagonists, while in fully developed flowers, anthocyanins may attract pollinators, and in fruits they may help to attract seed dispersers (Whittall and Strauss, 2006; Landi et al., 2015).

Intraspecific variation in floral and vegetative anthocyanin concentration within and among populations is common and often adaptive (del Valle et al., 2015; Berardi et al., 2016; Menzies et al., 2016). Some studies have shown that populations at low latitudes or in xeric environments may be characterized by small flower size and/or large UV absorbing floral patterns (Herrera, 2005; Lacey et al., 2010; Campbell and Powers, 2015; Koski and Ashman, 2015).

A decade of work suggests that climatic (and topographic) features may be drivers of intraspecific variation in floral color, pattern, and size (Schemske and Bierzychudek, 2007; Arista et al., 2013; Koski and Ashman, 2015; del Valle et al., 2015; Berardi et al., 2016). Collectively, this work suggests that, when examining geographic variation in these floral traits, we should expect a general pattern in which low latitude, high temperature, high solar UV, and low precipitation environments may select (either directly or indirectly) for increased petal anthocyanin production, larger UV-absorbing floral patterns, lower mean UV petal reflectance and/or smaller flowers. If large UV floral patterns confer a direct fitness advantage to individuals in high-UV environments by protecting developing pollen from abiotic stress, we would expect that under high-UV conditions pollen produced by individuals with larger UV floral patterns would perform better than pollen produced by individuals with smaller UV patterns.

To test these predictions, and to contribute toward an understanding of large-scale geographic patterns in these floral features, we quantified UV mean petal reflectance, the proportion of the petal occupied by the UV-absorbing nectar guide, petal area, and estimated floral anthocyanin concentration in greenhouse-raised populations representing eight wild populations of *Clarkia unguiculata* (Onagraceae) that span a wide latitudinal and climatic gradient (Supplement 1). We also tested the ability of high solar UV radiation to exert direct positive selection on UV nectar guide size, which has been demonstrated in only one other species to date (Koski and Ashman, 2015).

This study provides the first report of geographic variation in multi-spectral floral color and pattern in *C. unguiculata*, while also providing an experimental test of the putative protective role of UV absorption for pollen performance. In this study we examine greenhouse-raised populations representing eight wild populations of *C. unguiculata* to address the following questions: (1) Is intraspecific variation in floral color, pattern, and size geographically structured? (2) Which specific components of climate/geography (if any) are good predictors of these traits? (3) Does exposure to supplemental UV light during floral development influence floral color (anthocyanin production)? (4) Does UV nectar guide size serve a protective function? In other words, under high UV growing conditions, are flowers with larger UV nectar guides better able to protect their developing pollen from damaging abiotic stress? (5) Do the geographic patterns observed here reflect what we would expect based on the patterns described in previous research?

## Materials and Methods

### Study Species

*Clarkia unguiculata* (Onagraceae) is a bee-pollinated, primarily outcrossing, annual species native to California that exhibits tremendous variation in flower color, pattern, and size (Ivey et al., 2016). *C. unguiculata* is pollinated primarily by a set of solitary bee species (the ‘*Clarkia*’ bees) but are also visited by generalists such as *Bombus* spp. (MacSwain et al., 1973; Moeller and Geber, 2005). The “*Clarkia* bees” and *Bombus* are all in the order Hymenoptera. Hymenopteran insects are trichromats and observations from 43 species of Hymenoptera reveal maximal visual sensitivity at 340 nm (UV), 430 nm (blue), and 535 nm (green) (Chittka et al., 1999; Skorupski and Chittka, 2010; Dyer et al., 2012, 2015; Papiorek et al., 2016). Consequently, flower color in these wavelengths is of particular interest to studies of floral color evolution and geographic variation.

The red-purple color of the flowers of *C. unguiculata* is produced by three major anthocyanins: malvidin, cyanidin and delphinidin (Soltis, 1986). Malvidin 3,5-diglucoside appears to predominantly contribute to red-purple flower color in this species (Bowman, 1987).

The flowers of *C. unguiculata* are hermaphroditic and protandrous (i.e., anther maturity precedes stigma receptivity), and each flower bears two whorls of dimorphic stamens. The inner whorl is smaller, shorter and often white; the outer whorl is several millimeters longer, matures later, and bears anthers that range from dark red to purple (Lewis and Lewis, 1955; Peach and Mazer, 2019). We classified the developmental stages of the flowers of *C. unguiculata* into three distinct phases: male stage 1 (when the short anthers release their pollen), male stage 2 (when the long anthers release their pollen), and female (stage 3, when the stigma becomes receptive). Previous work has shown that floral sex stage is a significant source of variation in multiple floral traits in this species (Peach et al., 2020). We included floral sex stage as an independent variable in our statistical analyses (described below) so that we could control for its effects on floral phenotype when testing for and characterizing variation among populations. See Supplement 2 for a more detailed justification and description of this categorization.

### Greenhouse Study

Seeds from eight wild populations of *C. unguiculata* were collected and cultivated for this study. These eight wild populations span a latitudinal range of 33.46826°- 39.68256°, which includes almost the entire range of the species (*C. unguiculata* is endemic to California which spans a ∼32°-42° latitudinal range). Population locations are reported in Supplement 1. In 2015-2016, we sampled seeds from 35 maternal families per population. Seeds were placed in coin envelopes (one maternal family per envelope), which were stored in plastic zip-lock bags with silica desiccant in a dark refrigerator at 5°C until use. In Fall 2016, ten seeds per maternal family (35 maternal families × 8 populations) were germinated in agar in 10cm diameter Petri dishes. Petri dishes holding dormant seeds were placed in a dark refrigerator at 5°C for one week to promote germination. After germination, three seedlings per maternal family were randomly selected and planted individually in cone-tainers (20.32 cm in length × 3.81 cm diameter SC10 cone-tainers,^1^ Tangent, OR, United States) filled with a custom soil mixture (5:1:1 Sunshine Grow #4, sand, worm castings, and.14.17 grams Island Seed and Feed fertilizer per 3.62 kilograms of soil^2^). Cone-tainers were placed in racks in the greenhouses at UC Santa Barbara and bottom watered for the duration of the study. Plants were grown under controlled temperature (10–18°C nighttime temperature range and a 13–29°C daytime temperature range), and the temperature was logged hourly so that the statistical models used here (described below) could include temperature as an independent variable. The temperature sensor was suspended from the ceiling of the greenhouse, directly between the two rows of light treatments. It is possible that there were differences in temperature and light environment between the light treatments that were not accounted for directly here. Racks of cone-tainers were rotated (within their assigned light treatment) in the greenhouse every two weeks for the duration of the study.

### Experimental Design

**FIGURE 1:**
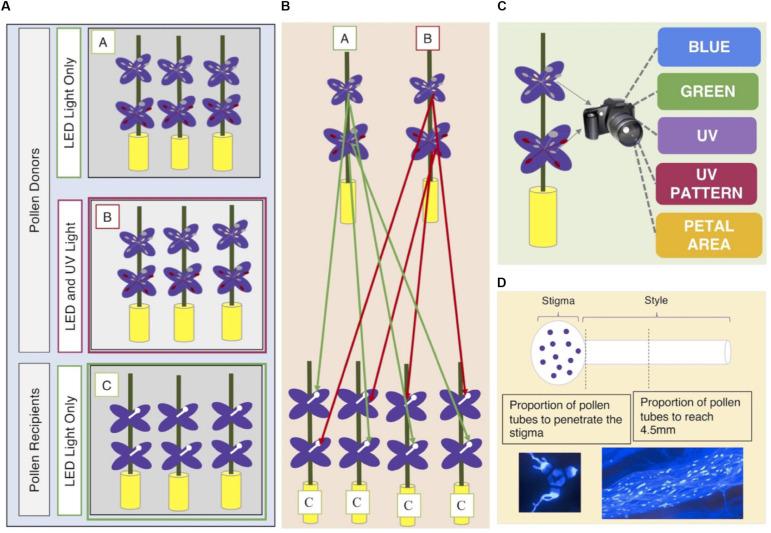
**(A)** Seeds from 35 maternal families per population were collected from eight wild populations of C. unguiculata. Three seedlings per maternal family were cultivated and assigned a group (A, B, C). Individuals in Group C were designated as pollen recipients. Pollen recipients (Group C) were grown under Lumigrow Pro LED lights for 10 h/day. Individuals in Groups A and B were designated as pollen donors. Pollen donors in Group A were also grown under Lumigrow Pro LED lights for 10 h/day. Pollen donors in Group B were grown under Lumigrow Pro LED lights and an additional full spectrum lamp (Exo Terro Sunray www.exo-terra.com) for 10 h a day. **(B)** We harvested pollen from both anther types from each pollen donor plant. From each pollen donor flower, we harvested pollen from the short set of anthers and, from another flower on the same plant, the long set of anthers to perform 1-3 hand pollinations (per set of anthers) on separate pollen recipient flowers (on separate pollen recipient plants). All hand pollinations were conducted using donors and recipients from the same population. **(C)** At the same time that we removed the anthers from each pollen donor flower we removed one petal. We took a multispectral image of the flower petal (see section “Materials and Methods”). **(D)** Four hours after each hand-pollination, we collected the stigma and style by severing the style at its base with a scalpel. We placed each stigma and style in a microcentrifuge tube filled with formalin acetic acid to arrest pollen tube growth. Staining with aniline blue and the use of a fluorescence microscope (with a DAPI filter) allowed us to visualize and count the pollen grains adhering to the stigma and the progress of pollen tubes down the style.

The experimental design is shown graphically in Figure 1. Each field-collected maternal family was represented by three siblings. The three siblings were separated into three groups (A, B, C). One sibling per family was designated as a ‘pollen recipient’ (individuals in Group C) and the other two as ‘pollen donors’ (individuals in Groups A and B). Pollen recipients (Group C) were grown under Lumigrow Pro LED lights (lumigrow.com) for 10 h a day for the duration of the study. As soon as the first flower opened on each individual pollen recipient, we removed both sets of anthers to prevent self-fertilization. Pollen recipients were physically separated from pollen donors in the greenhouse to prevent accidental cross-pollination (Figure 1).

The two pollen donor siblings were separated into two groups (A and B). Plants in group A were grown exclusively under Lumigrow Pro LED lights for 10 h a day. Plants in group B were grown under standard Lumigrow Pro LED lights and an additional UV lamp (Exo Terro Sunray^TM^,^3^) for 10 h a day. This additional lamp included light in the UV spectrum (300–400 nm) in addition to light in the visible spectrum. Plants in experimental light group B were exposed to both UV light and visible range light. We did not account for differences in the amount of visible light (green, blue, red light) between experimental groups A and B. In some species, UV radiation has been shown to be an important regulating factor of anthocyanin production and petal color formation (Llorens et al., 2015). The greenhouse glass filtered out the majority of UV light; however, it is likely that UV light was not 100% excluded from the individuals in Group A. We included light treatment (A or B) in our analyses for two reasons: first, to determine whether plants that were exposed to supplemental UV light produced more anthocyanins than their siblings that were exposed only to LED lighting; and, second, to determine the independent effects of supplemental UV light, anthocyanin concentration, and UV nectar guide size on pollen performance.

### Climate Data

To determine the effects of specific features of climate on flower color, pattern, and size, we extracted historic climate normals for each population using ClimateNA (Wang et al., 2016). This software extracts and downscales 1961-1990 monthly climate normal data from a spatial resolution of 4 × 4 km and calculates many monthly, seasonal and annual climate variables for specific locations based on latitude, longitude, and elevation (Wang et al., 2016). We extracted the mean values (from 1961 to 1990) of the following climatic variables for each population: mean temperature of the warmest month (C°), mean annual precipitation (mm), and summer heat moisture index. The summer heat:moisture index is a scaled, averaged (over a decade) ratio between mean warmest month temperature and mean summer precipitation. Hot, dry years/locations have relatively high indices, while cool, wet locations have relatively low indices.

For each site, we also extracted the sum of the solar radiation of the highest quarter (J/m^2^/day) for each population (Sum UV) from glUV, a global UV-B radiation dataset for macroecological studies (Beckmann et al., 2014), to use as a proxy for levels of solar UV radiation during the flowering period of *C. unguiculata*. glUV was generated using UV-B data from the Ozone Monitoring Instrument (OMI) onboard the NASA EOS Aura spacecraft (Beckmann et al., 2014). OMI contains two spectrometers and measures reflected solar radiation in a selected range of the visible (350–500 nm) and UV light spectrum (Levelt et al., 2006). The OMI measurements are used to calculate clear-sky surface UV irradiance, which is corrected for clouds and aerosols to obtain the OMI surface UV irradiance values (Tanskanen et al., 2006). The accuracy of these measurements, assessed with ground-reference data, ranges from 70% to 93%, depending on atmospheric and location-specific conditions (Tanskanen et al., 2006).

### Pollination Study

We harvested pollen from both anther types (see section “Study Species”) produced by each pollen donor in Groups A and B (*n* = 399). From each pollen donor flower, we harvested pollen from the short set of anthers and, from another flower on the same plant, the long set of anthers. These pollen samples were used to hand-pollinate 1-3 flowers (per set of anthers) on 1-3 separate pollen recipient flowers (see Figure 1). Each recipient flower received pollen from only one donor and one anther type. The inner (short) series of anthers produced only enough pollen to cover the surface of one stigma, so each set of short anthers was typically used in only one hand pollination. In all cases, the pollen recipients were from a different maternal family than the pollen donor plant. All hand pollinations were conducted using donors and recipients from the same population.

To perform each pollination, we selected the pollen donor flower and recorded the donor’s Individual ID number, floral sequence, anther type used, and light treatment. From this flower, we then removed one entire petal. We took a multispectral image of the petal using the method described below (see also Supplement 3). From the flower, we then removed the anthers of the type specified in the previous step and placed them in a microcentrifuge tube. Pollen grains were dislodged from the anther surface by agitating the closed microcentrifuge tube. We used a dissecting spatula to cover the stigmatic surface of an emasculated flower of the assigned pollen recipient with pollen from a single set of anthers. Four hours after each hand-pollination, we removed the stigma and style by severing the style at its base with a scalpel and placed each stigma and style in a microcentrifuge tube filled with formalin acetic acid to arrest pollen tube growth. Microcentrifuge tubes were labeled with an identification number linked to the pollen donor, pollen recipient, and light treatment of the associated sample. We also recorded the floral sequence of each pollinated flower on the primary stem of each pollen recipient. The floral sequence refers to the number of flowers produced before the focal flower, inclusive of the first flower (e.g., the first flower produced by a plant has a floral sequence of one). From among the total of ∼700 hand-pollinated flowers, pollen performance was unambiguously scored for 398 flowers (*n* = 230 pollinations using long-anther pollen and *n* = 168 pollinations using short-anther pollen).

Previous studies have shown that pollen germination rates and pollen tube growth rates are sensitive to temperature (Hedhly et al., 2009; Hove and Mazer, 2013; Mazer et al., 2018). Accordingly, we recorded the mean temperature of the greenhouse hourly during the four-hour window following each hand-pollination. For each flower, the mean greenhouse temperature recorded during the four-hour pollination window is included as an independent variable in the statistical analyses described below.

### Pollen Performance

We used the methods described by Martin (1959) to soften and clear the styles and to stain them with aniline blue. This process allows the visualization and counting of pollen grains and pollen tubes using a fluorescence microscope. A negative effect of pollen load on the proportion of pollen grains that penetrate the stigma due to gametophyte competition for either space or maternal resources has been demonstrated in *C. unguiculata* (Mazer et al., 2016). Therefore, to control for the potential effects of variation in the intensity of early-stage pollen competition on pollen performance, we counted the number of pollen grains adhering to each stigmatic surface and included this value as an independent variable in the models described below. Given that some of the pollen deposited on the stigma may have been dislodged when placed in solution in the microcentrifuge tube after they were harvested, the number of pollen grains adhering to the surface provides a measure of the number of grains that became anchored to the stigma within four hours of pollination (likely due to features of the pollen wall or germination) and are competing for access to the stigma. We quantified pollen performance by determining the proportion of these pollen grains that penetrated the stigma surface (PSP, proportion stigma penetrance) and the proportion whose tubes successfully grew to a distance of 4.5mm down the style (P4.5, the proportion of tubes to reach 4.5mm from the stigma surface) within the 4-hour post-pollination period. We selected 4.5mm because it represents ∼25% of the mean style length of this species (Peach and Mazer, 2019). We were able to quantify these metrics by examining the aniline blue stained stigma and style under a fluorescence microscope (with a DAPI filter) and counting the number of callose plugs deposited in the specified region of the pistil (cf. Hove and Mazer, 2013; Mazer et al., 2016; Mazer et al., 2018).

### Multispectral Photography and Image Analysis

When used properly, digital cameras are useful and relatively inexpensive tools with which to quantify color and pattern (Stevens et al., 2007; Spottiswoode and Stevens, 2010; Macfarlane and Ogden, 2012; Garcia et al., 2014; Troscianko and Stevens, 2015). Digital photography has been used in several studies to measure and to characterize plant and animal coloration, pattern, and camouflage (Strauss and Cacho, 2013; Stevens et al., 2017). In a related application of this approach, Del Valle et al. (2018) developed an efficient, non-invasive method for estimating anthocyanin concentration using red, green, and blue reflectance values extracted from digital images.

We used multispectral photography and image analysis to extract objective measurements of color-specific reflectance values and UV pattern information, petal area, and anthocyanin concentration of the flowers of *C. unguiculata.* Because digital cameras were initially designed to generate photographs for human vision, the sensors in “off the shelf” cameras typically come equipped to prevent ultraviolet (UV) and infrared light from hitting the sensor. This buffering can be removed, creating a “full spectrum” camera with a sensor that receives incoming light at all wavelengths. To quantify complex variation in color-specific reflectance, we converted a Panasonic LUMIX GX7 digital camera with its LUMIX 14-42mm II lens^4^ (Kadoma, Osaka Prefecture, Japan) to a ‘full spectrum’ camera using LifePixel conversion services^5^ (Mukilteo, WA, United States) and then analyzed the resulting images using an ImageJ software plug-in (originally created to characterize cuckoo eggshell patterns by Troscianko and Stevens, 2015). The camera lens used in this study transmits UV light that is received by the camera sensor.

There are other camera lenses (e.g., UV CoastalOpt^®^ SLR objective, or Nikon 105/4.5 UV Nikkor objective lens^6^) that are specifically designed to transmit UV light and that are capable of generating sharper UV images than the Lumix lens used here.

Given that our lens may not have transmitted 100% of UV light available, our measurements of UV mean petal reflectance may be underestimates. Recording absolutely accurate values of this trait (UV mean reflectance), however, was not required to address our primary questions, which required only that we obtain accurate relative values of UV mean petal reflectance within and between populations, which our camera provided. Consequently, the red, blue, and green reflectance measurements and UV nectar guide size reported here may all be interpreted as objective measures of petal color reflectance and size.

The Lumix GX7 comes with a Four Thirds (17.3 × 13 mm) CMOS sensor, which has a diagonal of 21.64 mm (0.85″) and a surface area of 224.90 mm^2^. Exposure and metering are easily manipulated in this camera model, which is required for unbiased data acquisition (Stevens et al., 2007). The Lumix GX7 is a mirrorless digital camera, which can calculate the optimal exposure under fixed lighting conditions using the “A” mode (aperture priority) setting (even in UV conditions) (Troscianko and Stevens, 2015). Once the optimal aperture was selected, the aperture setting remained fixed for the duration of the study and the camera automatically selected the best shutter speed. To ensure the capture of a perfectly exposed image, we used “exposure bracketing” whereby the camera automatically adjusts the exposure range across three photos to intentionally under- and over-expose the image by a set number of “exposure values”. Images were reviewed for acceptable exposure using the histograms available in the RawTherapee application.^7^ To calibrate each image to estimate petal area a ruler was included at a fixed position in every photograph. All images were captured in RAW format, which is required for reliable image extraction. RAW files contain unprocessed images which may be linearized using specialized software (The Multispectral Image Analysis Toolbox).

Both photos were taken at a station that included the full spectrum modified camera mounted on a tripod with a locked camera position and a standardized light source (a single 70 W Exo terro sunray, www.exo-terra.com, Mansfield, MA, United States). The height and the angle of the light source were not altered between photographs. We used two lens filters alternately to produce two images (which were later merged in ImageJ into a single multispectral image). The first image was created using a Baader U 2″ UV-pass filter (www.baader-planetarium.com, Mammendorf, Germany), which can be manually added to the camera to allow only ultraviolet (UV) light (300–400 nm with peak permeability at 350 nm) to hit the sensor, thereby generating a UV image. To create the second image, we replaced the UV-pass filter with a UV/Infrared light blocking filter^8^ (UVR Defense Tech, United States), which allows only visible green, blue and red light to pass through and hit the camera sensor.

We calibrated digital photographs to allow their use for objective measurements of color or pattern within or between photographs (Stevens et al., 2007). We used the “Multispectral Image Analysis Toolbox” (Troscianko and Stevens, 2015), which is a plugin for ImageJ (Schneider et al., 2012). Several studies have used this toolkit (Stevens et al., 2017; Gómez et al., 2018; van den Berg et al., 2019; Hawkes et al., 2019; Price et al., 2019; Šulc et al., 2019), which has many advantages including; easy linearization, high precision, and low data loss in image analysis due to 32-bit floating-point image processing (Troscianko and Stevens, 2015). The image frame included a fixed position ruler and Spectralon^TM^ diffuse reflectance standard with a flat reflectance value of 20% across the entire light spectrum, including red, blue, green, and UV wavelengths^9^ (North Sutton, NH, United States). For image calibration, we selected the Spectralon^TM^ diffuse reflectance standard (20% reflectance, Troscianko and Stevens, 2015) and used the setting for “visible + UV” photography and “aligned normalized 32-bit” files. This calibration process successfully linearized the RGB and UV values in each photograph.

The Multispectral Image Analysis Toolbox plugin (Troscianko and Stevens, 2015) for ImageJ (Schneider et al., 2012) combines red, green, blue, and UV wavelengths into one multispectral image and facilitates the extraction of objective measurements of color-specific reflectance values and UV pattern information. UV nectar guide area was determined by viewing the multispectral image in the UV channel and using existing ROI (Region of Interest) tools in ImageJ to draw a line along the border of the visually apparent UV-absorbing nectar guide in the claw of the petal. Objective measurements of mean UV (300–400 nm), blue (430–500 nm), green (510–530 nm) and red (560–580) reflectance were recorded for each petal analyzed (*n* = 700). We measured these wavelengths specifically due to their role in pollinator attraction (Peach et al., 2020) and to estimate anthocyanin concentration (see below). The area (mm^2^) of each region was also recorded. Image acquisition methods are described in detail in Supplement 3.

#### Estimation of Anthocyanin Concentration

Anthocyanins are present in most plant organs and are usually stored in vacuoles of epidermal cells (Lee and Finn, 2007; Gould et al., 2008; Landi et al., 2015). Anthocyanins absorb light at specific wavelengths, and the remaining light is reflected outward, which produces the visible colors in wavelengths of 400 – 700 nm (Kay et al., 1981; van der Kooi et al., 2016). When color-specific reflectance values (red, blue, green) are calculated for plant tissues using digital photography and multispectral image analysis (described above), there are several equations that can be used to estimate anthocyanin concentration without destructive sampling (Del Valle et al., 2018). Del Valle et al. (2018) found that color-specific reflectance values extracted from digital images in the visible light range can be used to accurately estimate anthocyanin concentration in floral tissues. We took multispectral images of flower petals to quantify their anthocyanin concentration using this method. To estimate anthocyanin concentration, we extracted only red, blue, and green reflectance values from “visible range” digital images. We did not use UV reflectance values to calculate anthocyanin concentration. We calculated anthocyanin concentration using the “S_green” equation described by Del Valle et al. (2018), which was reported by the authors to provide estimates of anthocyanin concentrations that are slightly more accurate than spectrophotometry. Total amounts of anthocyanins are reported as absorbance units (AU) per cm^2^ of fresh material.

#### Statistical Analysis

##### Geographic variation in petal color, pattern, and size

We calculated the population mean for each light treatment and functional sex stage. We treated each functional sex stage as distinct because in previous work we found that functional sex is a significant source of variation in these floral traits (Peach et al., 2020). Using these population means (8 populations × 2 light treatments × 3 functional sex stages = 48 mean values) of each of our focal floral traits as a dependent variable, we conducted several stepwise OLS (ordinary least squares) regressions to detect the effects of floral functional sex stage (male stage 1, male stage 2, and female [stage 3]), light treatment (LED vs. LED + UV), latitude, elevation, mean temperature of the warmest month (C°) (MTWM), mean annual precipitation (mm) (MAP), summer heat moisture index (SHMI), the sum of the solar radiation of the highest quarter (J/m^2^/day) (SumUV) and all two-way interactions. We calculated variance inflation factors for all of our bioclimatic predictor variables and identified problematic multicollinearity. Our goal was to identify the independent effects of each bioclimatic predictor on our focal floral traits, which we would be unable to do if collinear variables were included in the same model. We split the analyses of our bioclimatic predictors into two models, such that within each model there was no high collinearity between any of the predictor variables (all variance inflation factors of main effects were < 5). By splitting the analysis into two models, each model’s output avoided unreliable parameter estimation due to multicollinearity among its included variables. Variance inflation factors are reported with the parameter estimates in Supplement 4.

The predictor variables included in the final model were also used to construct an ANOVA (conducted using Type III sums of squares) to determine the independent effects of each predictor variable/interaction on each focal floral trait. We used Type III sums of squares (SS) to test for the effects of our bioclimatic predictors on each focal floral trait when each variable was placed last into the model; this tests for the effect of each variable independent of all others included in the model. Adjusted partial R^2^ values were calculated using the ‘rsq.partial’ function in the rsqv1.1 package in R studio.

##### Testing the protective function of UV floral patterns

We scored pollen performance within the 398 hand-pollinated flowers. Each data point in this analysis represents the pollen performance measurements from a single hand-pollinated flower (not a population mean). For each measure of pollen performance (PSP and P4.5), we conducted a stepwise OLS regression to detect the effects of anther type (long vs. short), light treatment, floral sequence, greenhouse temperature (during pollen germination and growth), anthocyanin concentration, proportion nectar guide, UV mean petal reflectance, and all two-way interactions. We conducted two additional stepwise OLS regressions (one for each pollen performance trait) to detect the effects of anther type, light treatment, floral sequence, greenhouse temperature, MTWM, MAP, SHMI, SumUV and all two-way interactions. We also constructed an ANOVA (conducted using Type III SS) to determine the independent effects of each predictor variable and interaction on pollen performance. All analyses were conducted in R Studio version 1.2.1335 (Lüdecke, 2019).

## Results

### Petal Color and Size

**FIGURE 2:**
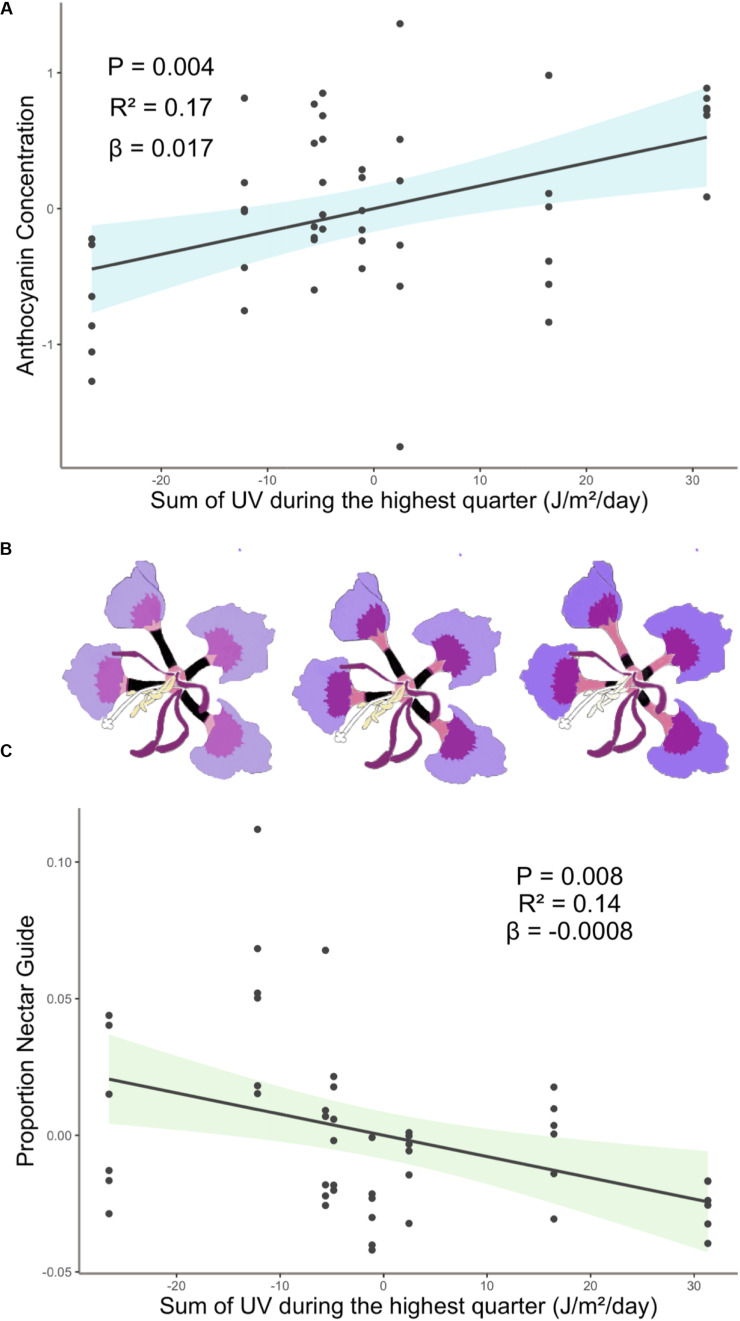
**(A)** Anthocyanin concentration was highest in populations with high SumUV **(B)** Representative depiction of geographic variation in estimated mean floral anthocyanin concentration and proportion nectar guide of eight populations of *C. unguiculata.* UV-absorbing nectar guides begin at the base of the claw and extend up each claw toward the blade. In this figure they are depicted as black rectangles, which is similar to how they appear in UV photos. **(C)** Proportion nectar guide was largest in low SumUV populations. Shaded area indicates the 95% confidence interval. β = the regression coefficient associated with the independent variable in each panel. *P* = the *P*-value associated with the regression coefficient. *R*^2^ = the partial r squared associated with the independent variable in each panel.

**FIGURE 3:**
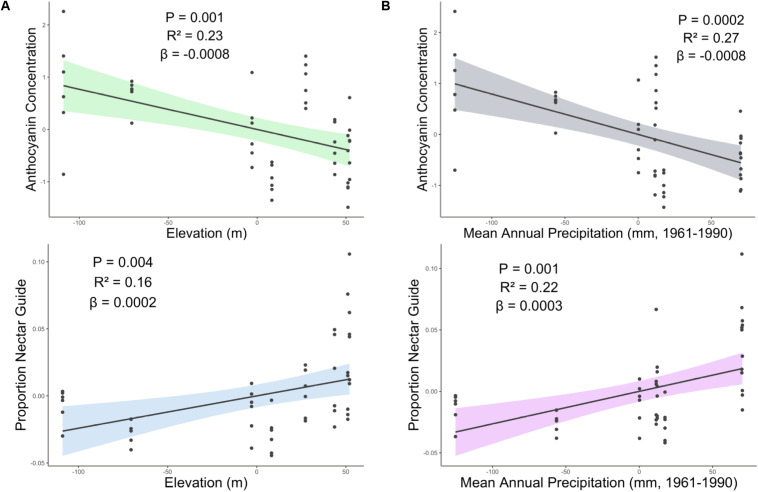
Partial regression plots derived from linear models showing the effect of **(A)** elevation and **(B)** precipitation on proportion nectar guide and anthocyanin concentration. Shaded area indicates the 95% confidence interval. β = the regression coefficient associated with the independent variable in each panel. *P* = the *P*-value associated with the regression coefficient. *R*^2^ = the partial r squared associated with the independent variable in each panel.

Experimental light treatment and several environmental variables had direct effects on petal color. Supplemental UV light (Light treatment B) had a significant negative effect on anthocyanin concentration but did not affect any other trait measured in this study (Table 1). Latitude and SumUV of the sampled populations’ locations had significant positive effects on anthocyanin concentration and mean UV petal reflectance (Table 1 and Figures 2, 3). The interaction between latitude and SumUV also had a significant effect on anthocyanin concentration and mean UV petal reflectance (Supplement 5). The slope of the relationship between SumUV and anthocyanin concentration was significant only at extreme latitudes (very low or very high). MAP and elevation had significant negative effects on anthocyanin concentration (Table 1 and Figure 3). Petal area (mm^2^) decreases with increasing latitude and SumUV (Table 1 and Figure 3). Model parameter estimates, and standard errors are reported in Supplement 4.

**TABLE 1:** **(a–c)** Summary of multivariate models to detect the independent effects of light treatment, floral sex stage, latitude, temperature, and solar UV on proportion nectar guide, anthocyanin concentration, mean UV petal reflectance, nectar guide area and petal area in *C. unguiculata*.

(a)	Proportion nectar guide				Anthocayanin concentration			
	SS	DF	*F*-value	Pr(>F)	SS	DF	*F*-value	Pr(>F)
(Intercept)	0.009	1	7.771	0.008	3.544	1	8.982	0.005
Light treatment	1.00E-04	1	0.096	0.758	3.428	1	8.689	0.005
Floral stage	0.004	2	1.893	0.164	5.541	2	7.022	0.002
Latitude	0.008	1	7.516	0.009	3.573	1	9.056	0.005
Mean temperature of the warmest month (°*C*)	0.007	1	6.776	0.013	0.202	1	0.512	0.478
Sum of the solar radiation of the highest quarter (J/m^2^/day)	0.008	1	7.746	0.008	3.667	1	9.296	0.004
Latitude:Sum of the solar radiation of the highest quarter (J/m^2^/day)	0.008	1	7.656	0.009	3.858	1	9.779	0.003
Model	0.04	40	2.367	0.04	15.781	40	11.3	< 0.0001
Adjusted R^2^				0.17				0.61

**(b)**	**Mean UV petal reflectance**				**Nectar guide area**			
	**SS**	**DF**	***F*-value**	**Pr(>F)**	**SS**	**DF**	***F*-value**	**Pr(>F)**

(Intercept)	3.849	1	11.153	0.002	9.099	1	15.76	0.0003
Light treatment	0.983	1	2.849	0.09	1.00E-05	1	0.00	0.997
Floral stage	0.625	2	0.905	0.413	8.888	2	7.697	0.001
Latitude	3.482	1	10.086	0.003	8.953	1	15.508	0.0003
Mean temperature of the warmest month (°*C*)	10.381	1	30.072	2.51	3.357	1	5.814	0.02
Sum of the solar radiation of the highest quarter (J/m^2^/day)	3.699	1	10.718	0.002	9.099	1	15.761	0.0003
Latitude:Sum of the solar radiation of the highest quarter (J/m^2^/day)	3.208	1	9.294	0.004	9.025	1	15.632	0.0003
Model	13.808	40	13.74	< 0.0001	23.094	40	5.915	0.0001
Adjusted R^2^				0.65				0.42

**(c)**	**Petal area**							
	**SS**	**DF**	***F*-value**	**Pr(>F)**				

(Intercept)	0.996	1	5.421	0.025				
Light treatment	0.02	1	0.11	0.742				
Floral stage	28.711	2	78.104	1.54E-14				
Latitude	0.943	1	5.132	0.029				
Mean temperature of the warmest month (°*C*)	0.225	1	1.224	0.275				
Sum of the solar radiation of the highest quarter (J/m^2^/day)	0.919	1	5.002	0.031				
Latitude:Sum of the solar radiation of the highest quarter (J/m^2^/day)	0.915	1	4.978	0.031				
Model	7.352	40	30.82	< 0.0001				
Adjusted R^2^				0.82				

**(d)**	**Proportion nectar guide**				**Anthocayanin concentration**			
	**SS**	**DF**	***F*-value**	**Pr(>F)**	**SS**	**DF**	***F*-value**	**Pr(>F)**

(Intercept)	0.0001	1	0.142	0.708	10.006	1	15.315	0.0003
Light treatment	1.00E-04	1	0.103	0.75	3.428	1	5.247	0.03
Floral stage	0.004	2	2.026	0.145	5.541	2	4.24	0.02
Mean annual precipitation (mm)	0.012	1	12.974	0.001	11.022	1	16.87	0.0002
Elevation (m)	0.009	1	9.161	0.004	8.811	1	13.486	0.0007
Mean annual precipitation (mm):Elevation (m)	0.01	1	10.657	0.002	10.193	1	15.601	0.0003
Model	0.039	41	3.258	0.01	26.789	41	5.156	0.0005
Adjusted R^2^				0.22				0.35

**(e)**	**Mean UV petal reflectance**				**Nectar guide area**			
	**SS**	**DF**	***F*-value**	**Pr(>F)**	**SS**	**DF**	***F*-value**	**Pr(>F)**

(Intercept)	0.132	1	0.26	0.613	10.474	1	16.103	0.0002
Light treatment	0.983	1	1.929	0.172	1.00E-05	1	0.00	0.997
Floral stage	0.625	2	0.613	0.547	8.888	2	6.832	0.003
Mean annual precipitation (mm)	0.467	1	0.917	0.344	11.303	1	17.377	0.0002
Elevation (m)	0.829	1	1.627	0.209	9.312	1	14.316	0.0005
Mean annual precipitation (mm):Elevation (m)	0.083	1	0.163	0.689	10.508	1	16.155	0.0002
Model	20.902	41	8.532	< 0.0001	5.21	41	26.668	0.0004
Adjusted R^2^				0.49				0.35

**(f)**	**Petal area**							
	**SS**	**DF**	***F*-value**	**Pr(>F)**				

(Intercept)	0.325	1	0.81	0.373				
Light treatment	0.02	1	0.05	0.823				
Floral stage	28.711	2	35.779	1.02				
Mean annual precipitation (mm)	0.403	1	1.004	0.322				
Elevation (m)	0.59	1	1.47	0.232				
Mean annual precipitation (mm):Elevation (m)	0.678	1	1.689	0.201				
Model	16.45	41	12.69	< 0.0001				
Adjusted R^2^				0.6				

### UV Floral Pattern

To our knowledge, this is the first study to describe the presence of UV-absorbing floral patterns (nectar guides) in *C. unguiculata*. Mean proportion nectar guide ranged from 0.05–0.57 (± SE 0.002, CV = 41.31) across populations and light treatments. Across *C. unguiculata*’s sampled range, latitude and SumUV had significant negative effects on the proportion nectar guide (latitude β = −0.343 ± SE = 0.125, SumUV β = −0.0008 ± SE = 0.0003) and nectar guide area (latitude β = −11.759 ± SE = 2.986, SumUV β = −0.026 ± SE = 0.007) (Table 1, Figure 1 and Supplement 4). This pattern was consistent using multiple measurements of UV including SumUV, mean summer solar radiation, and mean annual solar radiation. All three of these measurements of solar UV were strongly positively correlated, so only one (SumUV) was selected for the analyses presented here.

Among the populations examined here there was no correlation between anthocyanin concentration and proportion nectar guide (*P* = 0.87) or anthocyanin concentration and mean UV petal reflectance (*P* = 0.69). If the geographic patterns observed in this study were artifacts of a shared developmental pathway between UV-absorbing flavonoids and anthocyanin-flavonoids we would expect a strong negative correlation between these variables.

The interaction between SumUV and latitude had a significant effect on proportion nectar guide and nectar guide area. The slope of the relationship between SumUV and proportion nectar guide was significant only at relatively extreme latitudes. See Supplement 4 for a visualization of the effects of this interaction on UV floral patterns. MTWM, MAP and elevation had significant positive effects on proportion nectar guide and nectar guide area (Table 1, Supplement 4).

### Pollen Performance

**FIGURE 4:**
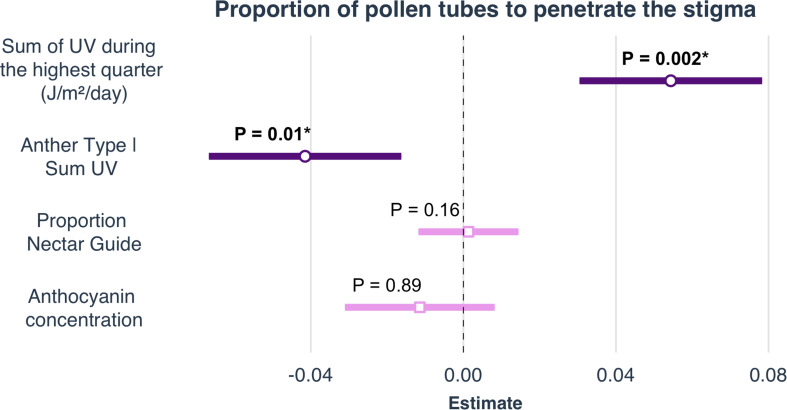
Selected parameter estimates of linear models to determine the effects of climate, latitude and elevation on proportion nectar guide, anthocyanin concentration, petal area, and mean UV petal reflectance. Estimates are visualized as forest plots (using the ‘plot_summs’ function in the jtools v2.0.1 package). The point within each bar is the estimate of the regression coefficient. The length of the bar represents the 95% confidence interval (CI) of this parameter.

Anther type, floral position, pollen load, greenhouse temperature, and environmental conditions of the sampled field sites all influenced pollen performance. Anthocyanin concentration and proportion nectar guide, however, did not influence pollen performance under either light treatment, independent of variation in the other variables included in the model (Table 2 and Figure 4). The anther type from which the pollen was collected had a significant effect on both PSP and P4.5. Pollen harvested from the shorter series of anthers performed better than pollen harvested from the longer series (PSP β = 0.019 ± SE = 0.008, P4.5 β = 0.044 ± SE = 0.012). The position of the pollen donor’s flower from which pollen was used had a significant positive effect on the PSP; pollen produced by relatively distal flowers (those with higher floral sequence values) had higher PSP than pollen produced by relatively basal flowers (β = 0.006 ± SE = 0.009). Pollen load had a significant negative effect on the PSP (β = −0.0002 ± SE = 0.00004, Table 2, Supplement 4). Mean greenhouse temperature did not have a significant effect on PSP or P4.5. Pollen performance also differed among populations sampled from environmentally distinct geographic regions. Among the field sites from which seeds were collected for this experiment, SumUV had a significant positive effect on PSP (β = 0.00003 ± SE = 0.00001) but no effect on P4.5. Parameter estimates for interaction terms are reported in Supplement 4.

**TABLE 2:** **(a)** Summary of multivariate models to detect the independent effects of anther type, greenhouse temperature, pollen load, floral sequence, SumUV and MTWM on pollen performance in *C. unguiculata*. **(b)** Summary of multivariate models to detect the independent effects of anther type, greenhouse temperature, pollen load, floral sequence, proportion nectar guide, anthocyanin concentration and mean UV petal reflectance on pollen performance in *C. unguiculata*. SS = Sum of squares.

(a)								
	Proportion of pollen tubes to penetrate the stigma (PSP)				Proportion of pollen tubes to reach 4.5 mm from the base of the stigma (P4.5)			
Term	DF	SS	F Ratio	Pr(>F)	DF	SS	F Ratio	Pr(>F)
Anther Type	1	0.12	6.567	0.011*	1	0.544	11.961	0.001*
Mean Greenhouse Temperature (°*C*)	1	0.383	20.888	<0.0001*	1	0.004	0.098	0.755
Anther Type ^∗^ Mean Greenhouse Temperature	1	0.489	26.71	<0.0001*	1	0.009	0.203	0.652
Pollen Load	1	0.647	35.296	<0.0001*	1	0.297	6.539	0.011*
Floral Sequence of the Pollen Donor	1	0.952	51.932	<0.0001*	1	0.056	1.232	0.268
Sum of the UV of the highest quarter (J/m^2^/day)	1	0.185	10.106	0.002*	1	0.083	1.823	0.178
Mean temperature of the warmest month (°*C*)	1	0.076	4.151	0.042*	1	0.199	4.369	0.037*
Anther Type^∗^ SumUV	1	0.113	6.139	0.014*	1	0.009	0.197	0.657
Model	8	3.315	22.61	<0.0001*	8	1.866	5.131	<0.0001*
Error	389	7.13			389	17.685		
C. Total	397	10.445			397	19.551		
Adjusted R^2^				0.3				0.08

**(b)**								

Anther Type	1	0.139	7.318	0.007*	1	0.618	13.178	0.0003*
Mean Greenhouse Temperature (°*C*)	1	0.226	11.895	0.001*	1	0.065	1.382	0.241
Anther Type ^∗^ Mean Greenhouse Temperature	1	0.372	19.588	<0.0001*	1	0.014	0.291	0.59
Pollen Load	1	0.577	30.376	<0.0001*	1	0.336	7.166	0.008*
Floral Sequence of the Pollen Donor	1	0.936	49.256	<0.0001*	1	0.003	0.068	0.794
Proportion Nectar Guide	1	0.041	2.173	0.141	1	0.011	0.242	0.623
Anthocyanin concentration	1	0.002	0.128	0.721	1	0.043	0.906	0.342
Mean UV Petal Reflectance	1	0.001	0.072	0.789	1	0.0199	0.425	0.515
Model	8	3.08	20.262	<0.0001*	8	1.319	3.513	0.001*
Error	386	7.333			386	18.116		
C. Total	394	10.413			394	19.435		
Adjusted R^2^				0.28				0.05

## Discussion

The tremendous diversity of floral form has held the interest and imagination of biologists for centuries. Evolutionary biologists frequently consider the diversification of flowering plants to be primarily driven by a combination of variation in abiotic (habitat and landform) and biotic (pollinator choice, availability and efficiency) conditions (Harder and Barrett, 2006; Rausher, 2008). Geographic variation in floral traits across heterogeneous landscapes provides the opportunity to examine the process and outcome of natural selection in the wild (Whittall and Strauss, 2006; Tripp et al., 2018).

The climate of North America will change dramatically over the coming decades, altering the selective pressures that influence the evolution of floral traits in wild populations (IPCC AR4, 2007; Christiansen et al., 2011). Understanding the mechanisms by which plants have responded to existing variation in climate can broaden our knowledge of evolutionary processes and inform estimates of how populations and species may respond to climate change (Harder and Barrett, 2006; Hoffmann and Sgrò, 2011.). The large geographic range of *Clarkia unguiculata* provides a rich mosaic in which to study the factors associated with the evolutionary diversification of floral traits beyond the traditional plant-pollinator framework.

### Is Intraspecific Variation in Floral Color and Pattern Geographically Structured in This Species?

In this study we found that intraspecific variation in floral color, pattern, and size is geographically structured and that several components of climate (and topography) explain a significant proportion of variation in these traits. However, only some of the geographic patterns reported here are consistent with those observed in other species (Koski and Ashman, 2015; del Valle et al., 2015; Berardi et al., 2016).

Latitude and elevation are often used as a proxy for a suite of abiotic conditions (Nybakken et al., 2004; Körner, 2007; Berardi et al., 2016; Bergamo et al., 2018). In this study we used long-term climate normals for several parameters to determine which climate features are correlated with flower pigment, pattern, and size in *C. unguiculata*. We included latitude and elevation in the models because they may serve as a proxy for one or more unmeasured factors. Pollinator assemblage or abundance, humidity, or herbivore/florivore pressure are all examples of factors that may be associated with latitude or elevation but were not accounted for directly here (Arnold et al., 2009; Jaakola and Hohtola, 2010; Tsuchimatsu et al., 2014; Berardi et al., 2016). For example, in some plant species, high anthocyanin concentration is associated with reduced herbivory (Irwin et al., 2003; Gould, 2004; Hanley et al., 2009). If herbivore pressure is more intense at lower elevations/latitudes and anthocyanin concentration deters herbivory in *C. unguiculata* (as it does in other species), then this may explain the negative correlation between elevation/latitude and anthocyanin concentration observed here. However, we do not have detailed records of herbivore assemblage or abundance for all of the locations examined in this study.

### Which Floral Traits Are Best Predicted by Components of Climate and Geography in *C. unguiculata*?

#### Anthocyanin Concentration

Flavonoids are ubiquitous plant secondary metabolites. The best-known examples are the characteristic red, blue, and purple anthocyanin pigments of plant tissues (Winkel-Shirley, 2001; Grotewold, 2006). Flavonoids are important secondary metabolites that help to protect plants against UV radiation and other forms of abiotic stress (Burns, 2015; Llorens et al., 2015). Given the well-documented increase in solar radiation (UV) toward the equator, plant flavonoid production is expected to increase with decreasing latitude (Jaakola and Hohtola, 2010; Llorens et al., 2015; Tripp et al., 2018). However, some studies have shown that anthocyanin flavonoids may exhibit the opposite geographic pattern (Hårdh and Hårdh, 1977; Lätti et al., 2008). Studies of *Vaccinium myrtillus* (northern bilberry) (Lätti et al., 2008) and *Fragaria virginiana* (wild strawberry) (Hårdh and Hårdh, 1977) report that populations at higher latitudes were characterized by higher concentrations of anthocyanin than those at lower latitudes.

Our results did not corroborate this expected pattern. Many of the factors that purportedly influence flower color and size are often tightly correlated with each other (e.g., locations at low latitudes often have high solar UV) making it difficult to identify the proximate agents of selection. The coast of California is characterized by a high density of coastal fog for several months of the year, resulting in a latitudinal gradient that includes unique combinations of climate, such as high-UV and high latitude (Torregrosa et al., 2016). Among the populations of *C. unguiculata* sampled here, those growing closer to the equator were not characterized by petals with a higher concentration of anthocyanin than those at higher latitudes. However, populations characterized by high UV conditions were associated with higher mean anthocyanin concentration than those in low UV conditions (Figure 2). The pattern observed in *C. unguiculata* is not consistent with previous research that has shown a significant negative effect of latitude on UV nectar guide in flowers (Koski and Ashman, 2015), but it does suggest that variation in solar UV may contribute to the evolution of anthocyanin production in *C. unguiculata*.

The association between latitude and floral pigment is sometimes associated with underlying climatic variables, such as temperature or precipitation (in addition to, or instead of UV radiation) (Schemske and Bierzychudek, 2001; del Valle et al., 2015; Bontrager and Angert, 2016). For example, in *Linanthus parryae*, periods of heat or drought favor pink- or purple-flowered individuals over white-flowered ones because associated anthocyanins in vegetative tissues enhance stress tolerance (Schemske and Bierzychudek, 2001; Harder and Barrett, 2006). Contrary to the patterns observed in *L. parryae*, among *C. unguiculata* populations, high mean temperature (MTWM) of the field site had no effect on mean anthocyanin concentration. However, as predicted, MAP had a significant negative effect on anthocyanin concentration among populations of *C. unguiculata*.

Elevation (and associated climatic variables) may also be associated with a cline in anthocyanin production (Berardi et al., 2016; Gray et al., 2018). Populations at higher elevations may evolve to produce more flavonoids as a response to increased UV radiation and/or increased herbivore pressure (Nybakken et al., 2004; Gould et al., 2008). For example, Berardi et al. (2016) found that populations of *Silene vulgaris* at higher elevations had more darkly pigmented calyces than those at lower elevations. However, in the current study, elevation had a significant negative effect on estimated anthocyanin concentration in *C. unguiculata*. Populations of this species generally occur at elevations < 1500 m, and the highest elevation population sampled for this study occurred at 1157 m (Lewis, 2012). It is possible that the elevation range of this species is not wide enough to result in local adaptation to elevation alone. Additionally, in some cases, populations at low elevations may be subject to higher levels of herbivory than high elevation sites (Gao et al., 2019), which could explain the relationship between anthocyanin concentration and elevation that we observed.

#### Petal Area

Several studies of population differentiation have shown that, within species, flowers may be smaller in locations characterized by high temperatures and low water availability (compared to more mesic locations) (Galen, 2000; Bull-Hereñu and Arroyo, 2009; Bontrager and Angert, 2016). This pattern can be explained by the observation that smaller flowers increase female fitness in xeric climates, because they lose less water than large ones (Galen, 2000; Carroll et al., 2001; Herrera, 2005). For example, Galen (2000) found that, under drought conditions, *Polemonium viscosum* (Polemoniaceae) plants with smaller flowers were more likely to survive and produce fruit (compared to plants that produced larger flowers). Consistent with this pattern, Bontrager and Angert (2016) detected a negative effect of historic mean annual temperature on mean petal length among populations of *Clarkia* pulchella. Similarly, Herrera (2005) examined nine populations of *Rosmarinus officinalis* (Lamiaceae) and found that flowers decreased in mass as the habitat became drier and hotter.

In sum, we may expect populations in hot, dry regions to be characterized by small flowers either as a plastic response to reduced resources or as a result of adaptive evolution. Geographic variation in flower size (that is associated with aridity) is of particular interest to evolutionary ecologists because it can give rise to mating system variation (Jonas and Geber, 1999; Runions and Geber, 2000; Button et al., 2012). In the current study, however, MAP and MTWM had no significant effect on petal area among the populations of *C. unguiculata*. Sum UV and latitude both had significant negative effects on petal area, which suggests that solar UV may influence the evolution of petal area more than temperature and rainfall in this species.

### Does Exposure to UVB Light During Floral Development Influence Flower Color, Pattern, or Size?

In addition to our examination of the effects of climate and geography on floral pigment, one of our research goals was to determine whether petal color (i.e., anthocyanin production) responds plastically to UV exposure. In some species, UV radiation has been shown to affect petal color formation (Ben-Tal and King, 1997; Llorens et al., 2015; Henry-Kirk et al., 2018), and petal color in roses intensifies (due to increased accumulation of anthocyanins) following exposure to UV (Maekawa et al., 1980; Henry-Kirk et al., 2018). Anthocyanins are also responsible for the UV-induced coloration of *Anigozanthos* spp. (Haemodoraceae) perianths (Ben-Tal and King, 1997). Furthermore, exposure to UV-B radiation at the pre-bud break stage can affect flower color. For example, a pre-bud treatment that blocked UV exposure decreased anthocyanin production in several species of *Malus* (Rosaceae), resulting in pink petals instead of red ones (Dong et al., 1998). In the current study, our predictions regarding the effects of elevated UVB on floral traits were not corroborated. Plants grown under LED lights (which produce only red and blue light) produced petals with higher estimated anthocyanin concentration than plants grown under LED + UV lights. It is possible that plants within the LED + UV light treatment group produced more non-anthocyanin flavonoids than those in the LED only group. The unexpected increase in anthocyanin production in the LED only group we observed may reflect (unmeasured) differences in non-anthocyanin flavonoids with a shared biochemical pathway. However, we only measured anthocyanin concentration in this study. All plants were grown in greenhouses that block most incoming UV light, although it is possible that the small amount of UV light that did pass through the greenhouse glass was sufficient to stimulate anthocyanin production in this species.

### Does UV Nectar Guide Size Serve a Direct Protective Function in This Species?

Previous work suggests that populations growing closer to the equator may produce larger UV-absorbing patterns than those from higher latitude populations (Koski and Ashman, 2015). It has been suggested that this pattern is evidence that Gloger’s rule — an ecogeographic “rule” stating that pigmentation of endothermic animals will increase from high-latitude to equatorial regions due to changing selective pressures including heat, humidity, predation and UV irradiance — may apply to plants as well. For example, populations of *Argentina anserina* (Rosaceae) growing closer to the equator are characterized by flowers with larger UV-absorbing bullseyes than populations at higher latitudes (Koski and Ashman, 2015). Koski and Ashman (2015) suggest that this pattern may be a general one in plants. Similar to *A. anserina*, *C. unguiculata* is characterized by within-species variation in UV-absorbing nectar guide size and its wild populations are distributed across a wide latitudinal range. In this study we found the populations of *C. unguiculata* growing closest to the equator are characterized by significantly larger UV-patterns (nectar guides) than those at higher latitudes. However, contrary to our predictions, we found that SumUV had a significant negative effect on proportion nectar guide and nectar guide area. Previous work has shown a significant positive relationship between nectar guide size and pollen receipt in this species (Peach et al., 2020). It is possible that the relationship between latitude and proportion nectar guide observed here reflects adaptation to variation in pollinator availability or assemblage across the state.

Koski and Ashman (2015) confirmed experimentally that high UV may exert direct positive selection on UV pattern size (or the proportion of the petal occupied by the UV-absorbing bullseye) in *A. anserina* by absorbing incoming UV light and preventing it from damaging developing pollen grains. We found no association between UV floral pattern size (of a pollen donor’s flower) and pollen performance (PSP and P4.5) under either high or low UV experimental light treatments. *A. anserina* is characterized by flowers that are more bowl-shaped and upward facing than the flowers of *C. unguiculata*. The floral morphology of *A. anserina* may result in more exposure of pollen to direct UV light and therefore stronger selection for floral features that offer pollen protection. Additionally, it is possible the UV nectar guides produced by *C. unguiculata* are not large enough to influence the UV environment of the flower. We conclude that this particular function of UV floral patterns may be restricted to species that grow closer to the equator than *C. unguiculata* or that have more reliably upward facing, or cup-shaped flowers.

We found that plants grown from seeds collected from high UV locations produced pollen that performed better than the pollen produced by plants from low UV locations. While UV floral patterns may not confer any direct fitness advantage (in terms of pollen protection) it appears that high UV populations may be characterized by plants that produce higher quality pollen anyway. The pollen of *C. unguiculata* is often itself pigmented (the pollen produced by the longer set of anthers is typically red-purple). Future studies should examine anthocyanin concentration of the anthers and pollen grains themselves to determine if these pigments offer a direct protective function in high UV environments (Koski and Galloway, 2018). The results presented here also corroborate previous reports of significant differences in pollen performance between the dimorphic anthers of *C. unguiculata* (Peach and Mazer, 2019).

## Conclusion

This study fills several gaps in our understanding of geographic patterns of floral color, pattern, and size. By measuring multiple components of floral pigment and pattern, we identified a more complex geographic pattern than we would have by examining UV floral pattern or anthocyanin concentration alone. Additionally, we examined the effects long-term climate normals on population mean genotype, rather than using latitude or elevation as a proxy for local climatic conditions, which improved our ability to detect the effects of specific climatic variables on our focal floral traits. We found that mean proportion nectar guide (the proportion of the petal occupied by the UV-absorbing nectar guide) was highest in low latitude, low-UV populations, which only partially supports our expectations, given the large-scale latitudinal patterns previously reported in *Argentina anserina* (Koski and Ashman, 2015). Mean floral anthocyanin concentration was highest in populations with high UV and low precipitation, which supports our predictions (del Valle et al., 2015). Anthocyanin concentration and proportion nectar guide did not influence pollen performance in either of the light treatments used in this study, indicating that these floral features did not provide a protective function for pollen germination or growth under the conditions examined here.

Abiotic factors contribute to floral diversity and broad-scale geographic patterns of floral color, pattern, and size (Schemske and Bierzychudek, 2001; Warren and Mackenzie, 2001; Coberly and Rausher, 2003; Arista et al., 2013; Koski and Ashman, 2015; Tripp et al., 2018) and identifying meaningful signatures of selection on flower color across large-scale environmental gradients is most informative when considering numerous interacting features of climate and topography.

## Data Availability Statement

The datasets generated for this study are available on request to the corresponding author.

## Author Contributions

KP and SM conceived the idea, designed the methods and statistical analyses, and led the writing of the manuscript and the interpretation of the results. KP and JL collected the data, participated in analytical discussions, and analyzed the data. All authors contributed critically to the drafts and gave final approval for publication.

## Conflict of Interest

The authors declare that the research was conducted in the absence of any commercial or financial relationships that could be construed as a potential conflict of interest.

## Footnotes

1 www.stueweandsons.com

2 www.islandseed.com

3 www.exo-terra.com

4 www.panasonic.com

5 www.lifepixel.com

6 https://www.jenoptik.us/

7 https://rawtherapee.com/

8 www.uvroptics.com

9 www.labsphere.com
