# Supplementary material for: Climate Predicts UV Floral Pattern Size, Anthocyanin Concentration, and Pollen Performance in Clarkia unguiculata
Source: Front Plant Sci. 2020 Jun 16;11:847. doi: 10.3389/fpls.2020.00847 (PMC7308548; doi:10.3389/fpls.2020.00847)

Supplementary Material

**Supplement 1:**

Locations and map of wild populations of *C.unguiculata* from which seeds were collected

| Population Name | Latitude | Longitude | Elevation (m) |
| --- | --- | --- | --- |
| Emerson Oaks Reserve | 33.46826 | 117.0727 | 445 |
| Evey Canyon | 34.16367 | 117.6826 | 704 |
| Matilija Creek | 34.51474 | 119.3809 | 506 |
| Wishon Drive | 36.18794 | 118.6714 | 1157 |
| Garrapata State Park | 36.45499 | 121.9179 | 475 |
| Bear Creek Rd. | 37.16563 | 122.018 | 651 |
| Auburn Recreation Area | 38.93301 | 121.0116 | 364 |
| Dark Canyon Rd | 39.68256 | 121.3909 | 811 |


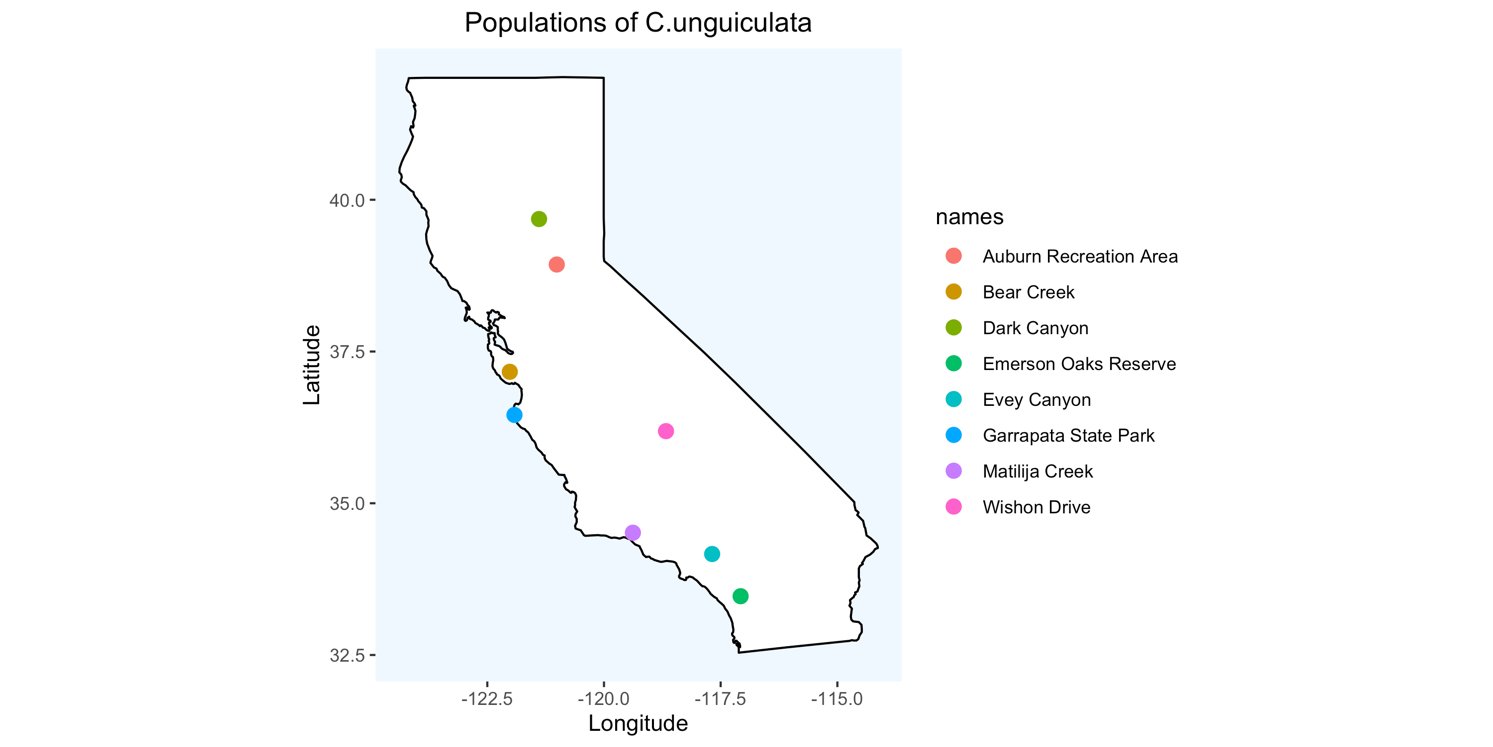


**Supplement 2:**

*Three distinct functional sex stages*

“We divided the male phase into two distinct stages because in *C. unguiculata* the inner whorl of anthers (stage 1) produces pollen with significantly higher performance than the outer whorl (Peach & Mazer, 2019). Heterantherous species such as *C. unguiculata* have been proposed to exhibit a ‘division of labor’ whereby ‘feeding anthers’ (which produce pollen that may be consumed by an insect) are distinguished from ‘reproductive anthers’ (which produce pollen more likely to contribute to reproduction) by differences in color and pollen performance (Mori et al., 1980; Nepi et al., 2003). Additionally, the petals of stage 1 (short anther maturity) flowers differ from those of stage 2 (long anther maturity) with respect to color, pattern and size (Peach et al., 2020) Because the two whorls of anthers in *C. unguiculata* may have evolved to serve distinct functions, we treated the dehiscence of each whorl of anthers as a distinct male stage.”

**References:**

**Mori SA, Orchard JE, Prance GT. 1980.** Intrafloral pollen differentiation in the New World Lecythidaceae, Subfamily Lecythidoideae. *Science* **209**(4454): 400–403. doi: 10.1126/science.209.4454.400

**Nepi M, Guarnieri M, Pacini E. 2003.** “Real” and feed pollen of *Lagerstroemia indica*: Ecophysiological differences. *Plant Biology* **5**(3): 311–314. doi: 10.1055/s-2003-40797

**Peach K, Mazer SJ. 2019.** Heteranthery in *Clarkia*: pollen performance of dimorphic anthers contradicts expectations. *American Journal of Botany*, **106**(4): 598– 603. <https://doi.org/10.1002/ajb2.1262>

**Peach K, Liu JW, Klitgaard KN, Mazer SJ. 2020.** Sex‐specific floral attraction traits in a sequentially hermaphroditic species. *Ecology and Evolution.*; 00: 1– 20. <https://doi.org/10.1002/ece3.5987>

**Supplement 3:**

*Multispectral Image Generation and Analysis-* We converted a Panasonic LUMIX GX7 digital camera with LUMIX 14-42mm II lens (www.panasonic.com, Kadoma, Osaka Prefecture, Japan) to a ‘full spectrum’ camera using LifePixel conversion services (www.lifepixel.com, Mukilteo, WA USA). After conversion, we used two lens filters alternately to produce two images (which were later merged in ImageJ). The first image was created using a Baader U 2” UV-pass filter (www.baader-planetarium.com, Mammendorf, Germany), which can be manually added to the camera to allow only ultraviolet (UV) light (300-400nm with peak permeability at 350nm) to hit the sensor, thereby generating a UV image. To create the second image, we replaced the UV-pass filter with a UV/Infrared light blocking filter (www.uvroptics.com, UVR Defense Tech USA), which allows only visible green, blue and red light to pass through and hit the camera sensor. The human red channel is partitioned out of the final multispectral photo such that pigment data from the red channel wavelengths are excluded from analysis.

Both photos were taken at a station which included the full spectrum modified camera on a tripod with a locked camera position and a standardized light source (a single 70W Exo terro sunray™, www.exo-terra.com, Mansfield, MA, USA). The height and the angle of the light source do not change between photographs. The image frame included a fixed position ruler and Spectralon™ diffuse reflectance standard with a flat reflectance value of 20% across the spectrum (www.labsphere.com, North Sutton, NH, USA). The reflectance standard is used in the ImageJ software to control for unavoidable variation in the lighting environment throughout the study (Stevens et al., 2007). The Multispectral Image Analysis Toolbox plugin (Troscianko and Stevens 2015) for ImageJ (Schneider et al. 2012) combines photos taken in the visible and ultraviolet spectrum (into one multispectral image) and facilitates the extraction of objective measurements of color-specific reflectance values and pattern. In all 32-bit multispectral images, each pixel has a numeric value between 1 and 65,535, which represented an objective measurement of wavelength-specific light reflectance. This value can be divided by 655.35 to attain a ‘percent reflectance’. We used this plugin to standardize and linearize the photographs and measure blue, green, and ultraviolet reflectance in different regions of the petal and determine the size of petal spots and nectar guides. A similar method of image acquisition and analysis was reported in detail earlier this year by Christian Verhoeven and colleagues (Verhoeven et al., 2018).

**References for Supplement 3:**

**Schneider CA, Rasband WS, Eliceiri KW. 2012.** NIH Image to ImageJ: 25 years of image analysis. Nature methods 9: 671-675.

**Stevens M, Párraga AC, Cuthill IC, Partridge JC, Troscianko J. 2007.** Using digital photography to study animal coloration. Biological Journal of the Linnean Society 90: 211–237.

**Troscianko J, Stevens M. 2015.** Image calibration and analysis toolbox – a free software suite for objectively measuring reflectance, colour and pattern. Methods in Ecology and Evolution 6: 1320–1331.

**Verhoeven C, Ren ZX, Lunau K. 2018** . False-colour Photography: A Novel Digital Approach to Visualize the Bee View of Flowers. Journal of Pollination Ecology 23: 102-118.

**Supplement 4:**

Parameter estimates and standard error for models summarize in Tables 1 and 2 (main document)

|  | **Proportion Nectar Guide** | | | | **Anthocyanin Concentration** | | | |
| --- | --- | --- | --- | --- | --- | --- | --- | --- |
| term | **Estimate** | **SE** | **t Ratio** | **Prob>\|t\|** | **Estimate** | **SE** | **t Ratio** | **Prob>\|t\|** |
| (Intercept) | 12.799 | 4.591 | 2.788 | 0.008 | -271.761 | 90.676 | -2.997 | 0.005 |
| Light Treatment A | 0.001 | 0.005 | 0.310 | 0.758 | 0.267 | 0.091 | 2.948 | 0.005 |
| Floral Stage 1 (male 1) | 0.009 | 0.006 | 1.432 | 0.160 | -0.054 | 0.128 | -0.417 | 0.679 |
| Floral Stage 2 (male 2) | 0.003 | 0.006 | 0.425 | 0.673 | 0.44 | 0.128 | 3.434 | 0.001 |
| Latitude | -0.343 | 0.125 | -2.741 | 0.009 | 7.428 | 2.468 | 3.009 | 0.005 |
| Mean temperature of the warmest month (℃) | 0.011 | 0.004 | 2.603 | 0.013 | 0.058 | 0.081 | 0.716 | 0.478 |
| Sum of the solar radiation of the highest quarter (J/m²/day) | -0.001 | 0.000 | -2.783 | 0.008 | 0.017 | 0.006 | 3.049 | 0.004 |
| Latitude : Sum of the solar radiation of the highest quarter (J/m²/day) | 0.000 | 7.489 | 2.767 | 0.009 | -0.0005 | 0.0001 | -3.127 | 0.003 |
|  | **Petal Area** | | | | **Nectar Guide Area** | | | |
| term | **Estimate** | **SE** | **t Ratio** | **Prob>\|t\|** | **Estimate** | **SE** | **t Ratio** | **Prob>\|t\|** |
| (Intercept) | 144.104 | 61.890 | 2.328 | 0.025 | 435.455 | 109.690 | 3.970 | 0.000 |
| Light Treatment A | -0.021 | 0.062 | -0.332 | 0.742 | 0.000 | 0.110 | -0.004 | 0.997 |
| Floral Stage 1 (male 1) | -1.029 | 0.088 | -11.757 | 0.000 | -0.606 | 0.155 | -3.910 | 0.000 |
| Floral Stage 2 (male 2) | 0.193 | 0.088 | 2.207 | 0.033 | 0.259 | 0.155 | 1.671 | 0.103 |
| Latitude | -3.817 | 1.685 | -2.265 | 0.029 | -11.759 | 2.986 | -3.938 | 0.000 |
| Mean temperature of the warmest month (℃) | -0.061 | 0.055 | -1.106 | 0.275 | 0.236 | 0.098 | 2.411 | 0.021 |
| Sum of the solar radiation of the highest quarter (J/m²/day) | -0.008 | 0.004 | -2.237 | 0.031 | -0.026 | 0.007 | -3.970 | 0.000 |
| Latitude : Sum of the solar radiation of the highest quarter (J/m²/day) | 0.000 | 0.000 | 2.231 | 0.031 | 0.001 | 0.000 | 3.954 | 0.000 |
|  | **Mean UV Petal Reflectance** | | | |  |  |  |  |
| term | **Estimate** | **SE** | **t Ratio** | **Prob>\|t\|** |  |  |  |  |
| (Intercept) | -283.252 | 84.817 | -3.34 | 0.002 |  |  |  |  |
| Light Treatment A | -0.143 | 0.085 | -1.688 | 0.09 |  |  |  |  |
| Floral Stage 1 (male 1) | -0.158 | 0.12 | -1.317 | 0.2 |  |  |  |  |
| Floral Stage 2 (male 2) | 0.107 | 0.12 | 0.896 | 0.38 |  |  |  |  |
| Latitude | 7.333 | 2.309 | 3.176 | 0.003 |  |  |  |  |
| Mean temperature of the warmest month (℃) | -0.416 | 0.076 | -5.484 | 2.51E+00 |  |  |  |  |
| Sum of the solar radiation of the highest quarter (J/m²/day) | 0.017 | 0.005 | 3.274 | 0.002 |  |  |  |  |
| Latitude : Sum of the solar radiation of the highest quarter (J/m²/day) | -0.0004 | 0.0001 | -3.049 | 0.004 |  |  |  |  |

|  | **Proportion Nectar Guide** | | | | **Anthocyanin Concentration** | | | |
| --- | --- | --- | --- | --- | --- | --- | --- | --- |
| term | **Estimate** | **SE** | **t Ratio** | **Prob>\|t\|** | **Estimate** | **SE** | **t Ratio** | **Prob>\|t\|** |
| (Intercept) | -0.021 | 0.055 | -0.377 | 0.708 | 5.71 | 1.459 | 3.913 | 0.000 |
| Light Treatment A | 0.001 | 0.004 | 0.321 | 0.750 | 0.267 | 0.117 | 2.291 | 0.030 |
| Floral Stage 1 (male 1) | 0.009 | 0.006 | 1.481 | 0.146 | -0.054 | 0.165 | -0.32 | 0.747 |
| Floral Stage 2 (male 2) | 0.003 | 0.006 | 0.440 | 0.662 | 0.44 | 0.165 | 2.668 | 0.011 |
| Mean annual precipitation (mm) | 0.000 | 0.000 | 3.602 | 0.001 | -0.008 | 0.002 | -4.11 | 0.000 |
| Elevation (m) | 0.000 | 0.000 | 3.027 | 0.004 | -0.008 | 0.002 | -3.67 | 0.001 |
| Mean annual precipitation (mm): Elevation (m) | -3.275 | 1.003 | -3.264 | 0.002 | 1.00E-05 | 2.64E-06 | 3.95 | 0.000 |
|  | **Mean UV Petal Reflectance** | | | | **Petal Area** | | | |
| term | **Estimate** | **SE** | **t Ratio** | **Prob>\|t\|** | **Estimate** | **SE** | **t Ratio** | **Prob>\|t\|** |
| (Intercept) | -0.657 | 1.289 | -0.510 | 0.613 | -1.029 | 1.143 | -0.900 | 0.373 |
| Light Treatment A | -0.143 | 0.103 | -1.389 | 0.172 | -0.021 | 0.091 | -0.225 | 0.823 |
| Floral Stage 1 (male 1) | -0.158 | 0.146 | -1.084 | 0.285 | -1.029 | 0.129 | -7.958 | 0.000 |
| Floral Stage 2 (male 2) | 0.107 | 0.146 | 0.737 | 0.465 | 0.193 | 0.129 | 1.494 | 0.143 |
| Mean annual precipitation (mm) | -0.002 | 0.002 | -0.958 | 0.344 | 0.002 | 0.002 | 1.002 | 0.322 |
| Elevation (m) | 0.002 | 0.002 | 1.275 | 0.209 | 0.002 | 0.002 | 1.213 | 0.232 |
| Mean annual precipitation (mm): Elevation (m) | 9.409 | 2.330 | 0.404 | 0.688 | 0.000 | 0.000 | -1.299 | 0.201 |
|  | **Nectar Guide Area** | | | |  |  |  |  |
| term | **Estimate** | **SE** | **t Ratio** | **Prob>\|t\|** |  |  |  |  |
| (Intercept) | -5.842 | 1.456 | -4.013 | 0.000 |  |  |  |  |
| Light Treatment A | 0.000 | 0.116 | -0.004 | 0.997 |  |  |  |  |
| Floral Stage 1 (male 1) | -0.606 | 0.165 | -3.684 | 0.001 |  |  |  |  |
| Floral Stage 2 (male 2) | 0.259 | 0.165 | 1.574 | 0.123 |  |  |  |  |
| Mean annual precipitation (mm) | 0.008 | 0.002 | 4.169 | 0.000 |  |  |  |  |
| Elevation (m) | 0.008 | 0.002 | 3.784 | 0.000 |  |  |  |  |
| Mean annual precipitation (mm): Elevation (m) | 0.000 | 0.000 | -4.019 | 0.000 |  |  |  |  |

|  | Proportion of pollen tubes to penetrate the stigma (PSP) | | | | | | | | Proportion of pollen tubes to reach 4.5mm from the base of the stigma (P4.5) | | | | | | | |
| --- | --- | --- | --- | --- | --- | --- | --- | --- | --- | --- | --- | --- | --- | --- | --- | --- |
| Term | **Estimate** | | **SE** | | **t Ratio** | | **Prob>\|t\|** | | **Estimate** | | **SE** | | **t Ratio** | | **Prob>\|t\|** | |
| Intercept | 0.129 | | 0.007 | | 18.026 | | 4.49E+00 | | 0.246 | | 0.011 | | 22.275 | | 3.71E+00 | |
| Mean Greenhouse Temperature (℃) | -0.033 | | 0.007 | | -4.557 | | 6.98E+00 | | 0.001 | | 0.014 | | 0.052 | | 0.958 | |
| Anther Type 1 | 0.019 | | 0.008 | | 2.452 | | 0.015 | | 0.044 | | 0.012 | | 3.684 | | 0.0003 | |
| Pollen Load | -0.0002 | | 4.00E-05 | | -5.753 | | 1.79E-08 | | 0.038 | | 0.012 | | 3.103 | | 0.002 | |
| Floral Sequence of the Pollen Donor | 0.006 | | 0.0009 | | 7.108 | | 5.77E+00 | | -0.007 | | 0.011 | | -0.662 | | 0.509 | |
| Sum of the UV of the highest quarter (J/m²/day) | 3.00E-05 | | 1.00E-05 | | 3.206 | | 0.001 | | 2.00E-05 | | 2.00E-05 | | 1.562 | | 0.119 | |
| Mean temperature of the warmest month (℃) | -0.018 | | 0.009 | | -1.967 | | 0.05 | | 0.026 | | 0.014 | | 1.866 | | 0.063 | |
| Mean Greenhouse Temperature (℃) * Anther Type 1 (short series) | -0.035 | | 0.007 | | -5.1609 | | 3.95E+00 | | -0.005 | | 0.013 | | -0.416 | | 0.677 | |
| Anther Type 1 *Sum of the UV of the highest quarter (J/m²/day) | 2.00E-05 | | 8.40E+00 | | 2.464 | | 0.014 | | 4.99E+00 | | 1.00E-05 | | 0.384 | | 0.702 | |
|  |  |  | |  | |  | |  | |  | |  | |  | |  |
|  | Proportion of pollen tubes to penetrate the stigma (PSP) | | | | | | | Proportion of pollen tubes to reach 4.5mm from the base of the stigma (P4.5) | | | | | | | |  |
| **Term** | **Estimate** | **SE** | | **t Ratio** | | **Prob>\|t\|** | | **Estimate** | | **SE** | | **t Ratio** | | **Prob>\|t\|** | |  |
| Intercept | 0.131 | 0.007 | | 18.293 | | 3.28E+00 | | 0.248 | | 0.011 | | 22.228 | | 5.81E+00 | |  |
| Mean Greenhouse Temperature (℃) | -0.027 | 0.008 | | -3.416 | | 0.001 | | 0.013 | | 0.012 | | 1.055 | | 0.292 | |  |
| Anther Type 1 | 0.021 | 0.008 | | 2.649 | | 0.008 | | 0.049 | | 0.012 | | 3.929 | | 0.0001 | |  |
| Pollen Load | -0.044 | 0.008 | | -5.52 | | 6.24E+00 | | 0.036 | | 0.012 | | 2.871 | | 0.004 | |  |
| Floral Sequence of the Pollen Donor | 0.05 | 0.007 | | 6.953 | | 1.54E+00 | | -0.001 | | 0.011 | | -0.064 | | 0.949 | |  |
| Mean UV Petal Reflectance | -0.002 | 0.008 | | -0.246 | | 0.806 | | 0.007 | | 0.012 | | 0.535 | | 0.593 | |  |
| Proportion Nectar Guide | -0.011 | 0.007 | | -1.464 | | 0.144 | | -0.006 | | 0.011 | | -0.533 | | 0.594 | |  |
| Mean Greenhouse Temperature (℃) * Anther Type 1 (short series) | -0.033 | 0.007 | | -4.429 | | 1.00E-05 | | -0.005 | | 0.011 | | -0.461 | | 0.645 | |  |
| Anthocyanin Concentration | -0.003 | 0.008 | | -0.414 | | 0.679 | | -0.008 | | 0.012 | | -0.614 | | 0.54 | |  |

Supplement 5.

The regression models described in the main text (results reported in Table 1, Figures 2 and 3) detected a significant effect of the interaction between SumUV and Latitude. To visualize the effects of this interaction term on our focal floral traits, we constructed Johnson-Neyman interval plots. In each case, the slope of the predictor (i.e., the y-variable in these figures) was statistically significant at only some values of the moderator (Latitude). The Johnson-Neyman interval provides the two values of the moderator (represented by the vertical dashed lines) at which the slope of the predictor changes from non-significant to significant. (**a**) When Latitude is < 36.61 or > 38.83 decimal degrees, the slope of SumUV (the effect of SumUV on proportion nectar guide) differs significantly from zero at p < .05. (**b**) When Latitude is <34.33 or > 36.97,  the slope of SumUV (the effect of SumUV on anthocyanin concentration)  differs significantly from zero at p < .05. (**c** ) When Latitude is <38.78, the slope of SumUV (the effect of SumUV on mean UV petal reflectance) differs significantly from zero at p < .05. (**d**) When Latitude is <36.86 or  >38.05, the slope of SumUV (the effect of SumUV on petal area) differs significantly from zero at  p < .05.


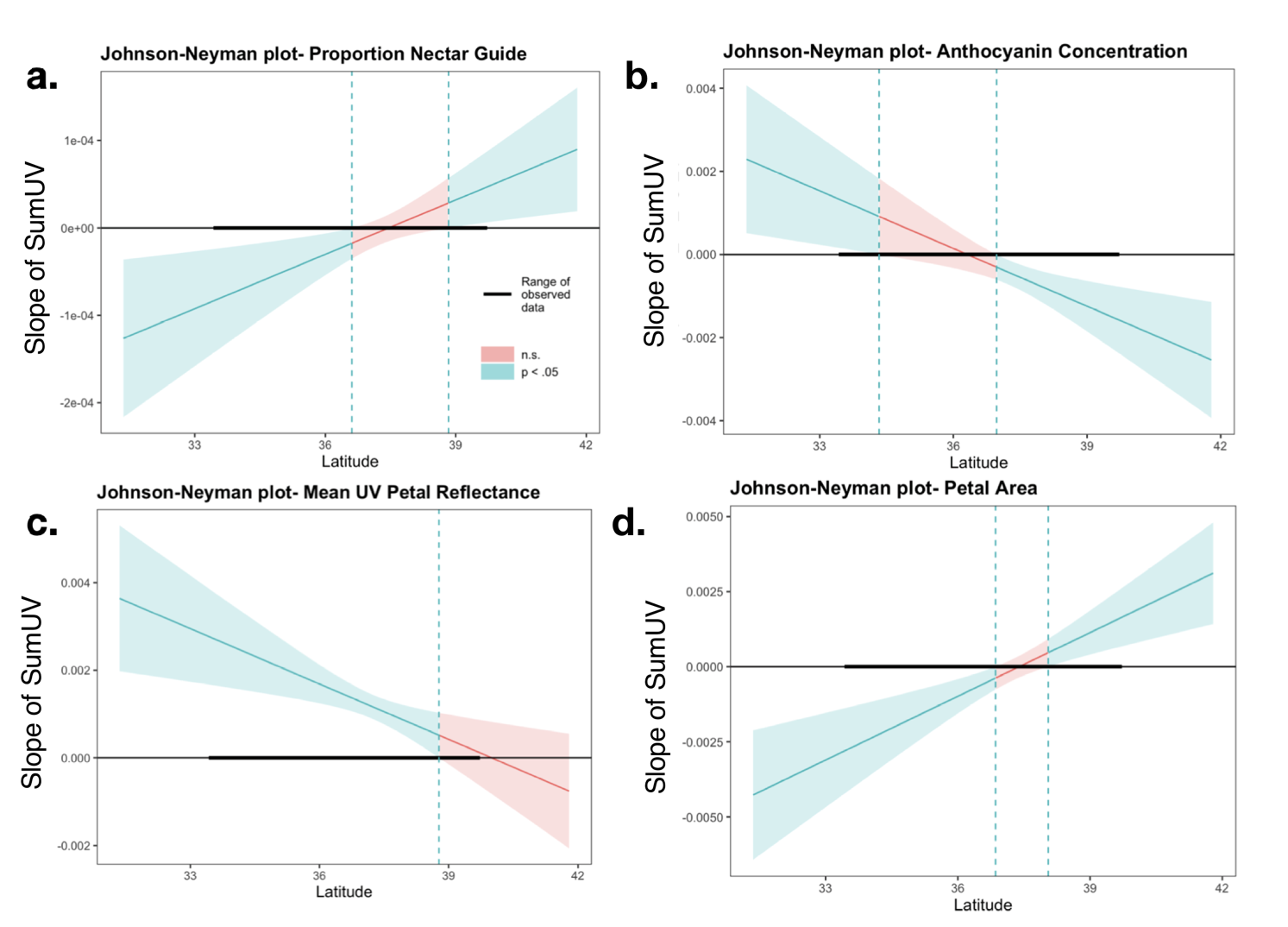

Supplement: Supplementary file 1 [file Data_Sheet_1.docx]
